# Special education teachers’ job demands-resources profiles and capabilities: Effects on work engagement and intention to leave

**DOI:** 10.3389/fpsyg.2022.942923

**Published:** 2022-10-13

**Authors:** Annelisa Murangi, Sebastiaan Rothmann, Mirna Nel

**Affiliations:** Optentia Research Unit, North-West University, Vanderbijlpark, South Africa

**Keywords:** job demands, job resources, capabilities, engagement, intention to leave, person-centered

## Abstract

This study aimed to investigate the job demands-resources profiles and work capabilities of special education teachers in Namibia and their effect on work engagement and intention to leave. A convenience sample was taken of teachers from seven different regions across Namibia (*N* = 200). The Capability Set for Work Questionnaire, the Job Demands-Resources Questionnaire, the Work Engagement Questionnaire, and the Intention to Leave Questionnaire were administered. Using latent profile analysis, four job demands-resources profiles were identified: resourceful job, demanding job, poor job, and rich job. A poor job was negatively associated with the capability to use knowledge and skills, while resourceful and rich jobs were associated with developing new knowledge and skills, being involved in important decisions, building and maintaining relationships, and setting own goals. Job experience was negatively associated with five of the seven capabilities. Resourceful and rich jobs and the capability set predicted a large percentage of the variance in work engagement and a moderate percentage of the variance in intention to leave. High emotional demands, coupled with overload and a lack of resources interfere with teachers’ functioning (e.g., work engagement and intention to leave).

## Introduction

Special education^1^ facilitates knowledge and skills acquisition for learners with disabilities. The work of special education teachers (SETs) is marked by individualized attention (Billingsley, 2004), where teachers must constantly strategize for the accommodation of learners with disabilities (Marfo et al., 2020). The competencies of SETs play a critical role in ensuring that learners receive quality teaching (Fauth et al., 2019), regardless of the type and nature of their disability (Allam and Martin, 2021). In African countries, where large inequalities exist, teachers face many challenges: a lack of teacher training, expertise and teaching materials, large class sizes, constraints on teacher time, and a lack of support (Chitiyo, 2006; Zemba and Chipindi, 2020). These contextual factors affect the work engagement (Murangi and Bailey, 2022) and retention of teachers (Billingsley, 2004; Thornton et al., 2007; Vittek, 2015). Research by Conley and You (2017) showed that one-third of novice SETs in the United States quit within the first 3 years of employment.

Disengagement and attrition of SETs are influenced by various factors within the teachers, the school setting, and the job (Major, 2012; Peyton et al., 2021). The job demands-resources (JD-R) model (Demerouti et al., 2001) has been developed to explain the work-related experiences of employees in terms of the balance between job demands and resources. High demands and a lack of resources contribute to SETs’ disengagement and decisions to leave (Conley and You, 2017). However, as Van der Klink et al. (2016) argue, individuals are more sustainably employable if their work is intrinsically valuable and does not only represent a way to earn a living. Therefore, deliberating values in the workplace and their enablement and achievement (i.e., capabilities) rather than an exclusive focus on employees’ subjective wellbeing is essential. A good balance between demands and resources is not enough for employees to function optimally; they also need a good balance regarding the values they consider relevant to their work (Van der Klink, 2019). The capability approach offers an appropriate framework that links teacher functionings (e.g., work engagement and intention to leave) to their capabilities (in terms of values, enablement, and achievement), job demands and resources, and conversion factors (Van der Klink et al., 2016).

The capability approach (CA; Sen, 1985), which was used to conceptualize the sustainable employability (SE) model (Van der Klink et al., 2016), integrates resources, context, and values to explain employees’ functionings. The SE model postulates that it is important to identify what people value in their work and if they can realize these valued aspects of work to support their sustainable employability (Van der Klink, 2019). Consequently, this model represents a shift in the thinking in occupational health psychology given its inclusion of ethical values (Van der Klink, 2019; Rothmann and Cooper, 2022).

Bakker et al. (2007, 2008) found that job resources (which balance high job demands) are good predictors of teachers’ work engagement, while high job demands, coupled with low job resources, lead to strain on employee wellbeing. Moreover, Bakker et al. (2003) showed that teachers who lack resources withdraw from work, which manifests as low motivation and commitment. Furthermore, Jackson et al. (2006), Janik and Rothmann (2015) confirmed that job demands and resources predict work-related functionings of teachers in South Africa and Namibia, respectively.

This study aimed to investigate the effects of job demands and resources on capabilities and functionings as conceptualized in the SE framework (see Van der Klink et al., 2016). Indeed, Billingsley and Bettini (2019) found that only a third of the studies on the retention of SETs used a conceptual framework to inform their research. Moreover, a meta-analytic review of longitudinal studies (Lessener et al., 2019) concluded that the JD-R model provided an excellent theoretical foundation for assessing employee wellbeing. However, research gaps exist regarding the capabilities of SETs and their association with job demands and resources and functionings (such as work engagement and intention to leave).

## The capability approach

### Conceptualization of capabilities

Functionings, capability, and agency are three key elements of the CA (Sen, 1988; Van der Klink et al., 2016). Functionings are a person’s beings and doings (i.e., states and activities). A person’s capabilities are the combinations of the functionings the person is able and enabled to achieve (Robeyns, 2017), considering resources and conversion factors (Van der Klink et al., 2016). The notion of agency pertains to the possibility of shaping one’s life and context and achieving valuable outcomes (Sen, 1988; Nussbaum, 2011). According to the CA, work should create value for employees and their place of work (Van der Klink et al., 2016), emphasizing what employees are effectively able to do or to be (their capabilities). Capabilities require individual freedom and agency to choose from a set of options about what constitutes a valuable life based on what is valuable to the individual employee (Walker and Unterhalter, 2007; Van der Klink et al., 2016).

The SE model (Van der Klink et al., 2016) emphasizes that, in addition to what employees value, it is essential to know whether they are able and enabled to achieve what they value. Capability for work refers to having the freedom to perform work that one values (Leßmann and Bonvin, 2011; Van der Klink, 2019). Employees’ sustainable employability becomes at risk when they cannot derive what they consider valuable from their work. According to Robeyns (2017), capability refers to a person’s ability or potential to achieve a functioning, i.e., the beings and doings that he or she values.

It is crucial to identify the capabilities of people (opportunities to achieve specific values, being able, and being enabled) in order to create a capability set for work Abma et al. (2016) argue that the capability set for work consists of seven non-ranked work values: using knowledge and skills, developing knowledge and skills, involvement in important decisions, meaningful contacts at work, setting own goals, earning a good income, and contributing to something valuable. These values can become capabilities if SETs find them important in their work, are enabled by contextual factors to achieve these values, and can achieve these values (Abma et al., 2016).

Developing capabilities requires resources and personal and social conversion factors (Robeyns, 2017). According to Leßmann and Bonvin (2011), job resources for work capability relate mainly to income and work conditions. However, capability for work also depends on personal and social conversion factors (Van der Klink et al., 2016). Personal conversion factors are skills and competencies. Social conversion factors comprise the number of available jobs, the accessibility to applicants, and job quality. The interdependence of resources and conversion factors makes it impossible to categorize them as resources or conversion factors (Leßmann and Bonvin, 2011). A person’s capability to convert available resources into valued capabilities is crucial since capabilities result from interactions between resources and conversion factors.

### Job demands and resources

#### The job-demands-resources model

Various job design and job stress theories have emphasized the role of either job demands or job resources in determining employees’ psychological states at work. The JD-R model investigates job demands and resources simultaneously (Demerouti et al., 2001), suggesting that job demands and resources are two sets of work conditions common to all jobs, regardless of the industry or occupation. The JD-R model examines how a lack of personal resources and imbalances between resources and job demands affect employee wellbeing (Lessener et al., 2019; Gabriel and Aguinis, 2022). According to Schaufeli and Taris (2014), there is no single JD-R model. Rather, the JD-R model represents an approach to how job characteristics (and personal characteristics) can affect workers’ wellbeing.

Job demands are “physical, social, or organizational aspects of the job that require sustained physical or mental effort and are therefore associated with certain physiological and psychological costs” (Demerouti et al., 2001, p. 501). For example, emotionally draining interactions or an intense workload and a high pace of work, emotional and physical pressure in work, role ambiguity, and role conflict associated with continuous changes in work are examples of job demands (Billingsley, 2004; Bakker, 2011; Minnotte, 2016). Job resources refer to those aspects of a job that are physically, psychologically, socially, or organizationally important for achieving work goals, reducing physical and psychological strain and stress, and stimulating personal growth and development (Bakker, 2011). For example, job autonomy, learning opportunities, supervisor support, remuneration, role clarity, and feedback are job resources (Rothmann et al., 2013; Minnotte, 2016; Lessener et al., 2019).

Job characteristics are a vital resource in the JD-R model. Although various job characteristics are regarded as job resources, autonomy and learning opportunities are critical (Rothmann et al., 2006; Gabriel and Aguinis, 2022). A job that provides employees with autonomy allows them to make their own decisions and control the tasks suited to their strengths and interests. Learning opportunities include projects or tasks (on and off the job) focused on developing employee skills. Employees with a broader skillset are more likely to manage and deal with job demands.

To optimize employees’ potential, supervisors must uncover the resources they need. For example, a supervisor may show concern for the wellbeing of employees by communicating that they are valued, encouraging them to set and achieve challenging, but achievable, new goals (Locke and Latham, 2020), and communicating their appreciation to them. In addition, supervisors need to involve employees in decision-making, particularly where their input is most valuable (Bashshur and Oc, 2015). Employees can influence work decisions through their feedback if they are involved in important decisions. In organizations, the term *voice* describes employees’ informal or formal expressions of ideas, opinions, and suggestions aimed at a specific target to change an objectionable situation and improve organizational functioning (Bashshur and Oc, 2015). Employees expect to feel appreciated by the supervisor, know their responsibilities and expectations, know what the supervisor thinks of their performance, and have the information they need about their work and how well they are doing (Rothmann et al., 2013).

To target employee performance, supervisors can link performance management to financial rewards (Gabriel and Aguinis, 2022). Remuneration and rewards are ways of engaging and motivating employees and making them feel that they are being treated fairly and justly. Performance feedback can be considered a valuable resource in the workplace (Gabriel and Aguinis, 2022). When employees receive clear, actionable feedback about their work, they can see the bigger picture, understand what they need to improve, and feel motivated to go the extra mile to achieve their goals (Rothmann et al., 2013; Gabriel and Aguinis, 2022). Performance feedback should focus on strengths and be timely, frequent, specific, verifiable, and consistent (Rothmann and Cooper, 2022).

Brunsting et al. (2022) found that a lack of autonomy, administrative support, and inadequate planning time predicted SET burnout of SETs. Minghui et al. (2018) showed that social support is a critical job resource for SETs. In Namibia, job resources, including autonomy, co-worker support, and rewards and recognition, showed moderate relationships with the work engagement of SETs (Murangi and Bailey, 2022).

Billingsley and Bettini (2019) found that SETs’ perceptions of job demands increased their intentions to leave, especially when these demands exceed their capacity to fulfill them. Concerning job resources of SETs, they established that administrative and collegial support contributed to the retention of SETs. Also, modest financial incentives contributed to lower intentions to leave (Billingsley and Bettini, 2019).

#### The effect of job demands and job resources on capabilities

Job demands might have adverse effects on capabilities depending on employees’ values. The same tasks can be performed from different perspectives. For example, on the one hand, an administrative task might be experienced as engaging by an employee with strong administrative skills who values knowledge and skills. On the other hand, such a task might burden an employee who values meaningful relations with people. Job autonomy and decision latitude as resources might play a role in converting emotional demands to values (e.g., developing new knowledge and skills, or contributing something valuable to society). However, they might also reflect capabilities such as involvement in important decisions and set their own goals (Van der Klink, 2019). Job autonomy might even be regarded as a precondition for other capabilities.

According to Sen (2009), resources such as income have value based on what people can accomplish and be when they use or convert them. Thus, resources are necessary, but not sufficient, for people to function optimally. Employees should have the possibility to take advantage of resources (Van der Klink, 2019). Employees can convert resources into opportunities through conversion factors instrumental to reaching valuable working goals (Robeyns, 2017). Van der Klink (2019) argues that conversion factors can clarify mechanisms that affect capabilities and functionings. For example, a good training and development policy might not have the intended effects if an organization lacks the staff to make it work. The conversion factors can also explain why people with ample resources do not actualize their full potential. Conversion factors that help employees achieve their valued outcomes despite difficulties are awareness of the organization, self-confidence, understanding of their abilities, and the willingness to compromise (Van Casteren et al., 2021). Brunsting et al. (2022) found that perceptions of workload manageability mediated the relationship between SETs’ working conditions and burnout.

#### Job demands-resources profiles: A person-oriented approach

Studies have often used a variable-oriented approach to study the associations between job demands and resources and the wellbeing of employees (Collie et al., 2020). According to Howard and Hoffmann (2017), variable-oriented approaches assume homogeneity of the population, which can provide important information about the patterns of relationships between samples and variables. However, this type of research cannot discern whether different subpopulations of employees experience the same demands and resources based on their common characteristics. A person-oriented approach, such as latent profile analysis, is ideal because it identifies distinct profiles of individuals with similar characteristics (Collie et al., 2020).

A person-oriented approach to job demands and resources is relevant to add to the literature on the CA and the JD-R model (Van den Broeck et al., 2011). Firstly, this approach can demonstrate the co-occurrence of job demands and job resources, thus helping to unravel the interrelationship between them. Secondly, a person-oriented approach enables the examination of capabilities and functionings that result from the combined effects of job demands and job resources throughout all job profiles. Thirdly, a person-oriented approach can indicate whether increasing job resources or decreasing job demands is more important for engagement and reducing employee intentions to leave. Using cluster analysis, Van den Broeck et al. (2011) reported four JD-R profiles: demanding, resourceful, poor, and rich jobs. They found that employees in demanding jobs (high job demands and low job resources) showed the poorest wellbeing.

Researchers have been studying teachers’ experiences at work from a person-oriented perspective (e.g., Collie et al., 2015; Collie and Martin, 2017). However, studies have not focused on JD-R profiles in special teaching contexts. Consequently, the association between such profiles and the capabilities and functionings of SETs remains unknown. Therefore, the distinct combinations of demands and resources for SETs in specific contexts must be investigated to inform policies and practices that facilitate capability identification, development, and optimal functioning. Understanding JD-R profiles is essential to promoting healthy and effective teachers and schools.

#### Functionings: Work engagement and intention to leave

Teacher functioning has implications for individuals and for the institutions (i.e., schools and educational institutions) that employ them. According to the CA, as previously mentioned, functionings refer to the beings and doings of individuals (Robeyns, 2017). Engagement and intention to leave are two critical teacher functionings.

According to Schaufeli et al. (2000), work engagement is a positive, fulfilling, work-related state of mind characterized by vigor, dedication, and absorption. Teacher engagement contributes to high levels of energy, which are essential for creativity in individualized special education to address diverse learning needs. Teachers who are enabled to capitalize on job resources such as supervisor support, co-worker support, and creative teaching resources can become engaged in their work. Dehaloo and Schulze (2013) found that teachers who experienced low levels of engagement expressed wishes for early retirement or resignation, increased absenteeism due to stress, poor involvement in the class, depression, and a lack of passion and dedication in their work, all of which contribute to poor teacher and learner performance.

Various researchers (e.g., Hagaman and Casey, 2018; Billingsley and Bettini, 2019) have expressed concerns about the retention of SETs. Despite an increased focus on why SETs leave the profession, research on teacher attrition is still lacking (Hagaman and Casey, 2018). Individuals’ intention to leave indicates the likelihood of them changing jobs within a specified period (Sousa-Poza and Henneberger, 2004). Literature reviews of special teacher education retention reveal that not enough knowledge of different disabilities, low job satisfaction, lack of professional development, overload, inadequate compensation, absence of mentorship programs, a negative school climate, lack of qualifications, lack of recognition and support from other teachers, and poor administrative support (Billingsley, 2004; Hagaman and Casey, 2018; Billingsley and Bettini, 2019) strongly affect teachers’ intention to leave and actual turnover. If SETs leave, the mandate of special education, which already functions on limited personnel capacity, can become compromised. Special schools cannot function sustainably without teachers, and learners cannot receive a quality education.

Employees who can achieve what they value in their work are more likely to be engaged and less likely to leave. Job demands (e.g., pace, workload, mental load, and emotional load) can facilitate or inhibit functioning. If demands are aligned with what employees value, they will be more engaged at work and less likely to leave (Van der Klink, 2019). Conversely, they may suffer from disengagement and leave if these demands are not valued.

### Current study

Namibian education authorities introduced the concept of special education in 1992, with the primary objective of helping children with disabilities acquire skills that will enable them to integrate into society (Republic of Namibia, Ministry of Education, 2018). All SETs in seven regions in Namibia (Erongo, Caprivi, Kavango, Khomas, Ohangwena, Oshana, and Omusati) formed part of the sampling population for this study. There is an estimated total of 300 SETs in Namibia. Teachers from 17 special schools, special classes in mainstream schools, and inclusive schools formed part of the sample for this study, because they teach learners with special needs (i.e., disabilities) in either educational option. As such, the term special education teacher was adopted in this study to refer to teachers who teach learners with disabilities in special schools, special classes in mainstream schools, and inclusive schools. Inclusive of private schools, Namibia has 1 184 schools, with an estimated student population of 755,943, of which around 24 005 are learners with disabilities enrolled at 17 special schools, special classes within mainstream schools, and inclusive schools around the nation^2^. In addition, an estimated 300 SETs are employed at these schools – fewer than mainstream education teachers. In Namibia, SETs receive a 4-year degree in Education, with an option to specialize in special education in the 4th year.

Despite advancements in policy, empirical research on special education teacher functioning in Namibia are scarce. Given the lack of resources in developing countries, teaching environments will inevitably have more demands and fewer resources due to vast inequalities. However, profiling SETs (through a person-oriented approach) can explain the naturally occurring patterns between job demands and resources and the number of teachers categorized in each profile. There is evidence of studies that investigated phenomena such as teacher engagement and wellbeing (Janik, 2013; Janik and Rothmann, 2015). However, these studies focused on mainstream schools (primary and secondary). Only one study (Murangi and Bailey, 2022) investigated engagement in SETs in Namibia related to job resources and job demands.

As in all variable-oriented approach studies, the studies mentioned above point to a relationship between one or two variables in a specific population. However, the person-oriented approach identifies whether subgroups exist within a specific population and uncovers the patterns that underlie such subgroups (Howard and Hoffmann, 2017). In this study, the person-oriented approach allowed in-depth profiling of individuals regarding their job demands-resources and capabilities and how such profiles could relate to their engagement and intention to leave. By categorizing SETs in different profiles, interventions can be tailored specifically for each profile.

The study of capabilities done by Abma et al. (2016) focused on the relation between the capability set for work and work role functioning, workability, work performance, hours worked, sickness absence, and sickness absence days in a Dutch working sample. It did not include work engagement and intention to leave as outcomes and correlates of the capability set for work, nor did it focus on SETs. This study aimed to identify JD-R profiles of SETs in Namibia and explore how distinct profiles were associated with their work capabilities and functionings (i.e., work engagement and intention to leave). In this study, latent classes for job demands and resources are not based on an *a priori* hypothesis. Latent profiles are generated and associated with capabilities and functionings. However, the following hypotheses were set for this study:

**Hypothesis 1:** Job demands-resources profiles are associated with the specific capabilities and capability set of SETs.

**Hypothesis 2:** Job demands-resources profiles and the capability set predict the work engagement of SETs.

**Hypothesis 3:** Job demands-resources profiles and the capability set predict the intentions to leave of SETs.

## Materials and methods

### Research design

The study took the quantitative approach by using a cross-sectional survey design. It is possible to draw conclusions about relationships among variables using cross-sectional designs, and it is also possible to eliminate possible alternative explanations for such relationships (Spector, 2019).

### Participants

All SETs in seven regions in Namibia (Erongo, Caprivi, Kavango, Khomas, Ohangwena, Oshana, and Omusati) formed part of the sampling population for this study. There is a total of 300 SETs in Namibia. Although 208 of the teachers responded to the survey, 200 responses were useable for this study.

As depicted in Table 1 (and considering that 1% of the participants did not indicate their gender), more female (68.5%) than male (30.5%) SETs participated in the study. In addition, 30.2% of participants obtained a degree as the highest qualification. Lastly, 98 participants (48.1%) had worked in the teaching profession for four to 13 years.

**TABLE 1:** Characteristics of participants (*N* = 200).

Demographic	Grouping	*N*	%
Gender	Male Female Missing values	61 137 2	30.5 68.5 1.0
Age group	20−30 years old 31−40 years old 41−50 years old 51−60 years old 60 + years old	49 69 44 22 16	24.5 34.5 22.0 11.0 8.0
Years of teaching experience	Less than 1 year 1 to 3 years 4 to 13 years 14 to 24 years 25 or more years Missing values	7 30 93 51 15 4	3.5 15.0 46.5 25.5 7.5 2.0
Years at current school	Less than 1 year 1 to 2 years 3 to 10 years 11 to 20 years 21 or more years Missing values	15 42 85 39 8 11	7.5 21.4 42.5 19.5 4.0 5.5
Highest teaching qualification	Grade 12 Diploma Postgraduate diploma graduate Degree Honors degree Master’s degree Missing values	19 47 34 59 19 15 7	9.5 23.5 17.0 29.5 9.5 7.5 3.5

### Measuring instruments

The Job Demands-Resources Scale (JD-RS; Rothmann et al., 2006) was utilized to measure job demands and resources. The JD-RS comprises 30 items about the pace and amount of work (three items, e.g., “Do you have too much work to do?”), mental load (three items, e.g., “Do you have to remember many things in your work?”), emotional load (three items, e.g., “Does your work put you in emotionally upsetting situations?”), opportunities to learn (two items, e.g., “Does your job offer you opportunities for personal growth and development?”), autonomy (four items, e.g., “Do you have freedom in carrying out your work activities?”), supervisor relationships (nine items, e.g., “Can you count on your supervisor when you come across difficulties in your work?”), remuneration (four items, e.g., “Do you think that your work pays good salaries?”), and career possibilities (two items, e.g., “Does your school give you opportunities to follow training courses?”). The items were rated on a five-point scale, ranging from 1 (*never*) to 5 (*always*). Cronbach’s alpha ranged from 0.76 to 0.92 (Rothmann et al., 2006), indicating acceptable reliability.

The Capabilities for Work Questionnaire (CWQ; Abma et al., 2016) measured capabilities. The CWQ measures three capability components: work values, enablement, and achievement. The seven values are as follows: (a) use of knowledge and skills; (b) development of knowledge and skills; (c) involvement in important decisions; (d) building and maintaining meaningful contacts at work; (e) setting own goals; (f) earning a good income; and (g) contributing to something valuable. For each valued aspect, respondents were asked whether (a) they thought this aspect was important to them (seven items, e.g., “How important is it for you to have or to be able to build meaningful working relationships at work?”), (b) their work offered them sufficient opportunities to do it (seven items, e.g., “Does your current work offer you enough opportunities to do that?”), and (c) they were able to succeed in realizing it (seven items, e.g., “To what extent do you succeed in doing so?”). Response options ranged from 1 (*not at all*) to 5 (*very much so*).

Three Flourishing at Work Scale items (FAWS; Rothmann et al., 2019a,b) were used to measure work engagement. The three items measure three dimensions of work engagement, for example, “At my work, I feel bursting with energy” (vigor), “I am enthusiastic about my job” (dedication), and “I am immersed in my work” (absorption). Schaufeli et al. (2017) reported that a scale using three items to measure work engagement shared 86−92% of its variance with a longer nine-item version. Also, the pattern of correlations between work engagement and other indicators was close for a three-item measure compared to a nine-item measure of work engagement. Rothmann et al. (2019b) found an acceptable reliability coefficient (ω = 0.85) for the scale.

The Turnover Intention Scale (TIS; Sjöberg and Sverke, 2000) was used to measure SETs intentions to leave. The TIS consists of three items (e.g., “If I were completely free to choose, I would leave this job”). Response options ranged from 1 (*strongly disagree*) to 5 (*strongly agree*). Moller and Rothmann (2019) validated the TIS in a study using managers from agribusinesses in South Africa and obtained a Cronbach’s alpha coefficient of 0.83. The TIS could, therefore, be relied on to measure intention to leave.

### Research procedure

Permission to conduct the research was sought from Namibia’s Ministry of Education, Arts, and Culture and its regional directorates. The researchers applied for ethical clearance from the North-West University Economic and Management Sciences Research Ethics Committee (EMS-REC) and was granted clearance (NWU-00840-20-A4). The researcher could only commence data collection after permission had been granted by the various regional directors and the EMS-REC. Participants were aware that the study was strictly voluntary and that they had an option to withdraw from the research process, at any given time, without incurring any negative consequences. The researcher assured participants of the confidentiality and anonymity of their data.

Teachers had to work from home because of the COVID-19 pandemic restrictions in Namibia, which delayed the data collection phase. Therefore, the study employed the use of an online and a hard-copy (printed) survey. The hard-copy surveys yielded a significantly high response rate of 96%.

### Data analysis

The data analysis for this study was done using SPSS27 (IBM Corporation, 2021) and Mplus 8.7 (Muthén and Muthén, 1998-2022). Several goodness-of-fit indices and information criteria were used to assess the fit of models (West et al., 2012): the chi-square statistic (the test of absolute fit of the model), standardized root mean residual (SRMR), root mean square error of approximation (RMSEA), Tucker-Lewis index (TLI), and comparative fit index (CFI). For TLI and CFI values to be acceptable, scores higher than 0.90 are required, while values larger than 0.95 indicate excellent fit. Both RMSEA and SRMR values lower than 0.08 indicate a close fit between the model and the data (Wang and Wang, 2020).

Latent profile analysis (LPA) was used to analyze different JD-R profiles using Mplus 8.7 (Muthén and Muthén, 1998-2022; Wang and Wang, 2020). The maximum likelihood with robust standard errors (MLR) estimator in Mplus was utilized. Different models with various latent profiles were tested utilizing the MLR estimator. A model was retained when a significant improvement was found from the reference model to the model with more profiles. Bayesian information criterion (BIC), Akaike information criterion (AIC), and sample-size adjusted Bayesian information criterion (ABIC) values were used to compare models (Kline, 2016; Wang and Wang, 2020). The optimal number of profiles was determined using the Lo-Mendell-Rubin test (LMR LR; Lo et al., 2001), the adjusted Lo-Mendell-Rubin test (ALMR), and the bootstrapped likelihood ratio test (BLRT; Wang and Wang, 2020). Entropy was verified to determine the quality of profile verification in LPA. Entropy values range from 0 to 1, with values closer to 1 indicating suitable classification (Geiser, 2013). The average latent profile probabilities were studied to determine the probability of correct class membership. When individuals are assigned to specific latent profiles, a probability value higher than 0.80 is generally considered a good indicator (Geiser, 2013).

Pearson correlation coefficients were used to determine the relationship between capabilities, engagement, and intention to leave (Field, 2013). Crosstabulation and Cramér’s V were used to determine the relationship between job demands and job resources, the capability set, work engagement, and intention to leave. Logistic regression analysis was performed on the seven capabilities as binary outcomes and JD-R profiles. Multiple regression analyses were employed to investigate the effects of JD-R profiles and capabilities on work engagement and intentions to leave.

## Results

### Confirmatory factor analysis

Confirmatory factor analysis (CFA) was used to assess the fit of the measurement model of job demands and job resources, work engagement, and intention to leave. The following fit statistics were obtained: χ^2^ = 976.187 (*df* = 566; *p* = 0.001), CFI = 0.94, TLI = 0.94, RMSEA = 0.06 [0.054, 0.067, *p* = 0.005], SRMR = 0.08. The sizes of the factor loadings of the items on their target factors were acceptable (see Table 2). Therefore, the factors were well-defined and corresponded to *a priori* expectations.

**TABLE 2:** Factor loadings on the latent variables.

Variable	λ (Range)	λ (Mean)
Overload	0.62−0.67	0.65
Emotional load	0.60−0.79	0.71
Task characteristics	0.65−0.86	0.75
Supervisor relations	0.57−0.86	0.75
Remuneration	0.89−0.92	0.91
Performance support	0.79−0.83	0.81
Work engagement	0.75−0.81	0.78
Intention to leave	0.77−0.93	0.86

### Descriptive statistics, reliabilities, and correlations

The means, standard deviations, omega reliabilities, and Pearson correlations of the variables in the current study are reported in Table 3. Reliability coefficients above 0.70 were obtained for all scales in the study, indicating acceptable reliability (Nunnally and Bernstein, 1994).

**TABLE 3:** Descriptive statistics, reliabilities, and correlations of the scales.

Variable	ω	Mean	*SD*	1	2	3	4	5	6	7	8
1. Workload	0.76	3.86	0.68	−	−	−	−	−	−	−	−
2. Emotional load	0.72	2.88	0.99	0.36**	−	−	−	−	−	−	−
3. Task characteristics	0.82	4.02	0.72	0.05	−0.11	−	−	−	−	−	−
4. Supervisor relations	0.89	3.84	0.80	−0.04	−0.19*	0.66**	−	−	−	−	−
5. Remuneration	0.93	2.55	1.21	−0.04	−0.03	0.20**	0.33**	−	−	−	−
6. Performance support	0.73	3.38	1.21	−0.04	−0.13	0.35**	0.52**	0.48**	−	−	−
7. Capability set	0.77	4.20	2.20	−0.02	0.00	0.51**	0.54**	0.33**	0.40**	−	−
8. Work engagement	0.76	5.05	0.76	0.08	−0.12	0.41**	0.37**	0.05	0.18*	0.34**	−
9. Intention to leave	0.89	2.28	1.06	0.07	0.14*	−0.31**	−0.18*	−0.21**	−0.15*	−0.26**	−0.19**

*p < 0.05; **p < 0.01.

Table 3 shows that the capability set was statistically significantly associated with task characteristics and supervisor relations (both large effects) and with remuneration, performance support, and work engagement (all medium effects). The capability set was also statistically significantly related to intention to leave. Task characteristics were statistically significantly and positively related to work engagement and negatively related to intention to leave (both medium effects). Supervisor relations were also statistically significantly and positively related to work engagement (medium effect).

Relationships between the capabilities, work engagement, and intention to leave were identified using point biserial correlations. Concerning work engagement, the correlations (*p* < 0.01) were as follows: use of knowledge and skills (*r* = 0.21), development of knowledge and skills (*r* = 0.22), involvement in important decisions (*r* = 0.24), building and maintaining meaningful relationships at work (*r* = 0.30), setting own goals (*r* = 0.22), earning a good income (*r* = 0.21), and contributing to something valuable (*r* = 0.24).

Regarding intention to leave, the correlations with capabilities were as follows: use of knowledge and skills (*r* = −0.27, *p* = 0.001), development of knowledge and skills (*r* = −0.19, *p* = 0.008), involvement in important decisions (*r* = −0.22, *p* = 0.002), building and maintaining meaningful relationships at work (*r* = −0.18, *p* = 0.010), setting own goals (*r* = −0.09, *p* = 0.212), contributing to something valuable (*r* = −0.14, *p* = 0.045), and earning a good income (*r* = −0.24, *p* = 0.001).

### Latent profile analysis

Latent JD-R profiles were analyzed using factor scores saved from the measurement model. Measurement error was controlled for by giving greater weight to items with smaller measurement errors (Morin et al., 2016). Table 4 presents the results of the five JD-R profiles.

**TABLE 4:** Comparison of different job demands-resources latent profile analysis models.

Profile	AIC	BIC	ABIC	LMR LR test *P*-value	ALMR LR test *P*-value	BLRT *P*-value
Profile 1	2515.09	2554.67	2516.65	n/a	n/a	n/a
Profile 2	2270.60	2333.26	2273.07	0.004**	0.005**	0.000**
Profile 3	2171.82	2257.58	2175.21	0.039*	0.042*	0.001**
Profile 4	2125.57	2234.41	2129.87	0.162	0.170	0.001**
Profile 5	2097.58	2229.52	2102.79	0.545	0.553	0.001**

AIC, Akaike information criterion; BIC, Bayesian information criterion; ABIC, adjusted Bayesian information criterion; LMR LR, Lo-Mendell-Rubin test; ALMR LR, adjusted Lo-Mendell-Rubin test; BLRT, bootstrapped likelihood ratio test.

**p < 0.01; *p < 0.05.

Table 3 shows that Profile 2 fitted the data better than Profile 1: ΔAIC = −244.49; ΔBIC = −221.41; ΔABIC = −243.58, LMR LR (*p* = 0.004), ALMR (*p* = 0.005), and BLRT (*p* < 0.001). Profile 3 fitted the data better than Profile 2: ΔAIC = −98.78; ΔBIC = −75.69; ΔABIC = −97.86, LMR LR (*p* = 0.039), ALMR (*p* = 0.042), and BLRT (*p* < 0.001). Furthermore, Profile 4 fitted the data better than Profile 3 on some of the fit indices: ΔAIC = −46.25; ΔBIC = −23.17; ΔABIC = −45.34, and BLRT (*p* < 0.001). Although Profile 5 showed slightly better fit indices than Profile 4, for example, ΔAIC = −27.99; ΔABIC = −27.08, and BLRT (*p* < 0.001), too few participants were placed in the profile.

**FIGURE 1:**
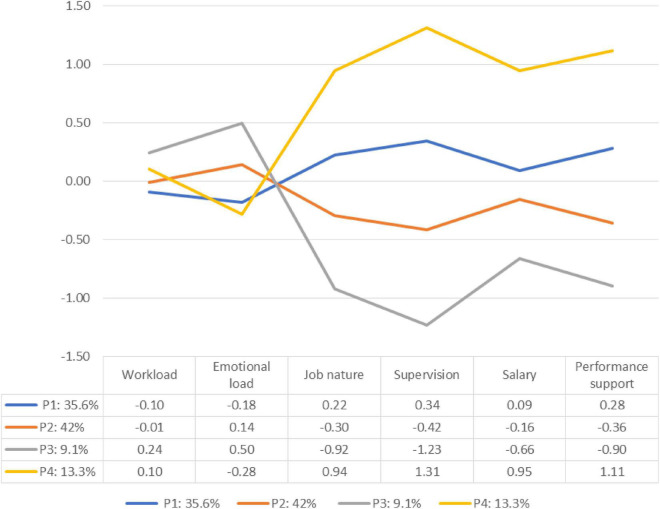
Latent profiles of job demands and resources of special education teachers.

The four latent profiles are shown in Figure 1. A total of 35.1% (*n* = 69), 42.5% (*n* = 85), 9.5% (*n* = 19), and 13.5% (*n* = 27) of the participants were assigned to Profiles 1, 2, 3, and 4, respectively. The proportions of participants in the four profiles were not too small. The average latent class probabilities were as follows: 0.92 (Profile 1), 0.91 (Profile 2), 0.89 (Profile 3), and 0.94 (Profile 4). The entropy value was 0.86, which represents a good classification (Wang and Wang, 2020).

In line with the four profiles identified by Van den Broeck et al. (2011), the profiles in Figure 1 can be interpreted as follows: (a) Profile 1: resourceful job (low job demands, moderate job resources). SETs in this profile experienced relatively low job demands and moderate to high job resources, although they experienced a moderate score on remuneration. (b) Profile 2: demanding job (moderate job demands, low job resources). Teachers in this profile experienced moderate workload and emotional load, and low job resources, indicating a concern regarding their job characteristics, relationships with their supervisor, remuneration, and performance support. (c) Profile 3: poor job (high job demands, low job resources). Teachers in this profile experienced very high demands. However, because of inadequate job resources, these teachers did not have the support to deal with the high job demands in their work effectively. (d) Profile 4: rich job (moderate demands, high resources). SETs in this profile had a moderate workload and a low emotional load. However, these teachers obtained high scores on all the job resources.

The following associations between the JD-R profiles and capabilities were obtained using Cramér’s V (φ; after computing crosstabulations): use of knowledge and skills (φ = 0.23), development of knowledge and skills (φ = 0.33), involvement in important decisions (φ = 0.42), building and maintaining meaningful relationships at work (φ = 0.42), setting own goals (φ = 0.36), earning a good income (φ = 0.41), and contributing to something valuable (φ = 0.35).

Tests of the three predictors against a constant-only model were statistically significant for the use of knowledge and skills, development of new knowledge and skills, involvement in important decisions, meaningful work relationships, setting own goals, earning a good income, and contributing to something valuable, indicating that the predictors significantly distinguished between capable and non-capable SETs.

### Regression analyses

#### Logistic regression analyses

A direct logistic regression analysis was performed on the seven capabilities as outcomes and JD-R profiles (see Table 5 for a summary of the results).

**TABLE 5:** Binary logistic regression analyses with job demands-resources profiles as independent variables and capabilities as dependent variables.

Variable	χ^2^	*df*	*R* ^2^	Percentage predicted			HL test	Significant predictors
				CC	CNC	Overall		
UKS	10.04*	3	0.07	94.7	17.6	68.5	NS	Poor job [Wald = 5.15*, OR = 0.30 (0.11, 0.85)]
DKS	22.64**	3	0.15	95.2	17.6	66.5	NS	Resourceful job [Wald = 7.99**, OR = 2.72 (1.36, 5.44)]; Rich job [Wald = 7.81**, OR = 5.11 (1.63, 15.05)]
IID	37.85**	3	0.23	66.3	71.9	68.5	NS	Resourceful job [Wald = 12.22**, OR = 3.27 (1.68, 6.34)]; Rich job [Wald = 16.30**, OR = 13.94 (3.88, 50.07)]
MRW	38.22**	3	0.24	97.7	22.5	70.5	NS	Resourceful job [Wald = 6.80**, OR = 2.55 (1.26, 5.17)]; Rich job [Wald = 8.73**, OR = 9.64 (2.14, 43.30)]; Demanding job [Wald = 6.43**, OR = 13.94 (0.04, 0.53)]
SOG	25.20**	3	0.16	21.2	96.3	71.5	NS	Resourceful job [Wald = 6.69**, OR = 2.62 (1.26, 5.43)]; Rich job [Wald = 5.27*, OR = 3.83 (1.22, 12.07)]; Demanding job [Wald = 6.43**, OR = 0.24 (0.08, 0.72)]
EGI	37.95**	3	0.23	27.8	98.2	66.5	NS	Rich job [Wald = 16.07**, OR = 21.77 (4.83, 98.22)]
CSV	27.41**	3	0.17	96.0	18.4	66.5	NS	Demanding job [Wald = 4.48*, OR = 0.30 (0.10, 0.92)]; Rich job [Wald = 9.49**, OR = 1.18 (2.36, 47.60)]

UKS, use of knowledge and skills; DKS, development of knowledge and skills; IID, involvement in important decisions; MRW, building and maintaining meaningful relationships at work; SOG, setting own goals; EGI, earning a good income; CSV, contributing to something valuable; CC, correct prediction of capable; CNC, correct prediction of not capable; HL test, Hosmer and Lemeshow test; NS, not significant.

*p < 0.05; **p < 0.01; R^2^, Nagelkerke R^2^; OR (odds ratio) = Exp(B).

The odds ratio of the statistically significant JD-R profiles as predictors of specific capabilities showed the following results: (a) a poor job is associated with a lower capability to use knowledge and skills; (b) resourceful and rich jobs are associated with a higher capability to develop new knowledge and skills; (c) resourceful and rich jobs are associated with a higher capability to be involved in important decisions; (d) resourceful and rich jobs are associated with a higher capability to build and maintain meaningful relationships, while a demanding job is associated with a lower capability to build and maintain meaningful relationships; (e) resourceful and rich jobs are associated with a higher capability to set their own goals, while a demanding job is associated with a lower capability to set their own goals; (f) a rich job is associated with a higher capability to earn a good income; (g) a demanding job is associated with a lower capability to contribute to something valuable, while a rich job is positively associated with this capability. Based on these findings, hypothesis 1 is partially accepted.

Logistic regression analyses using Mplus 8.7 showed that when age, job tenure (i.e., years in the current position), years in teaching, years at the school and JD-R profiles (as independent variables) were entered into the regression equation, only job tenure (together with the above-mentioned JD-R profiles) negatively predicted the following capabilities: use of knowledge and skills (β = −0.41, *p* = 0.005); development of knowledge and skills (β = −0.32, *p* = 0.017); involvement in important decisions (β = −0.48, *p* < 0.001); earning a good income (β = −0.35, *p* = 0.018); and contributing to something valuable (β = −0.53, *p* < 0.001).

#### Multiple regression analyses

Multiple regression analyses were used to investigate the effects of the JD-R profiles and capabilities on work engagement and intention to leave. Dummy variables were created for the different JD-R profiles using Profile 2 (demanding job) as the comparison group. Dummy variables are applicable when categorical variables have more than two categories (Field, 2013). Therefore, dummy variables make it possible to represent groups of people using only zeros and ones, which makes it possible to use categorical variables in regression analysis. The results of the multiple regression analyses are presented in Table 6.

**TABLE 6:** Multiple regression analyses of the job demands-resources profiles and capabilities on work engagement and intention to leave.

Model	Variable	Work engagement							Intention to leave						
		Beta	*SE*	ß	*p*	*R* ^2^	*F*	*p*	Beta	*SE*	ß	*P*	*R* ^2^	*F*	*p*
Model 1	Resourceful job	0.37	0.09	0.27	0.000**	0.25	21.98 *df* (3, 196)	0.000**	0.02	0.11	0.01	0.870	0.10	7.16 *df* (3, 196)	0.000**
	Poor job	–0.11	0.14	–0.05	0.436				0.28	0.17	0.12	0.097			
	Rich job	0.92	0.13	0.49	0.000**				–0.54	0.14	–0.27	0.000**			
Model 2	Resourceful job	0.37	0.09	0.27	0.000**	0.27	17.97 *df* (4, 195)	0.000**	0.02	0.10	0.02	0.814	0.14	8.21 *df* (4, 195)	0.000**
	Poor job	–0.06	0.14	–0.03	0.689				0.18	0.16	0.08	0.262			
	Rich job	0.89	0.12	0.47	0.000**				–0.49	0.14	–0.25	0.001**			
	UKS	0.19	0.09	0.14	0.031*				–0.31	0.10	–0.22	0.002**			
Model 1	Resourceful job	0.37	0.09	0.27	0.000**	0.25	21.98 *df* (3, 196)	0.000**	0.02	0.11	0.01	0.870	0.10	7.16 *df* (3, 196)	0.000**
	Poor job	–0.11	0.14	–0.05	0.436				0.28	0.17	0.12	0.097			
	Rich job	0.92	0.13	0.49	0.000**				–0.54	0.14	–0.27	0.000**			
Model 2	Resourceful job	0.35	0.09	0.26	0.000**	0.26	16.82 *df* (4, 195)	0.000*	0.06	0.11	0.04	0.588	0.11	6.25 *df* (4, 195)	0.000**
	Poor job	–0.09	0.14	–0.04	0.530				0.24	0.17	0.10	0.155			
	Rich job	0.89	0.13	0.47	0.000**				–0.48	0.15	–0.24	0.001**			
	DKS	0.98	0.09	0.07	0.263				–0.18	0.10	–0.13	0.071			
Model 1	Resourceful job	0.37	0.09	0.27	0.000**	0.25	21.98 *df* (3, 196)	0.000**	0.02	0.11	0.01	0.870	0.10	7.16 *df* (3, 196)	0.000**
	Poor job	–0.11	0.14	–0.05	0.436				0.28	0.17	0.12	0.097			
	Rich job	0.92	0.13	0.49	0.000**				–0.54	0.14	–0.27	0.000**			
Model 2	Resourceful job	0.36	0.10	0.26	0.000**	0.25	16.53 *df* (4, 195)	0.000**	0.07	0.11	0.05	0.508	0.12	6.34 *df* (4, 195)	0.000**
	Poor job	–0.10	0.14	–0.05	0.474				0.25	0.15	0.11	0.137			
	Rich job	0.89	0.13	0.47	0.000**				–0.44	0.15	–0.22	0.004**			
	IID	0.55	0.09	0.04	0.531				–0.19	0.10	–0.14	0.060			
Model 1	Resourceful job	0.37	0.09	0.27	0.000**	0.25	21.97 *df* (3, 196)	0.000**	0.02	0.11	0.01	0.870	0.10	7.16 *df* (3, 196)	0.000**
	Poor job	–0.11	0.14	–0.05	0.436				0.28	0.17	0.12	0.097			
	Rich job	0.92	0.13	0.49	0.000**				–0.54	0.14	–0.27	0.000**			
Model 2	Resourceful job	0.33	0.09	0.24	0.000**	0.27	17.97 *df* (4, 195)	0.000**	0.05	0.11	0.03	0.672	0.11	5.82 *df* (4, 195)	0.000**
	Poor job	–0.03	0.15	–0.01	0.834				0.22	0.17	0.10	0.200			
	Rich job	0.85	0.13	0.45	0.000**				–0.49	0.15	–0.25	0.001**			
	MRW	0.20	0.09	0.15	0.030*				–0.14	0.11	–0.10	0.192			
Model 1	Resourceful job	0.37	0.09	0.27	0.000**	0.25	21.98 *df* (3, 196)	0.000**	0.02	0.11	0.01	0.870	0.10	7.16 *df* (3, 196)	0.000**
	Poor job	–0.11	0.14	–0.05	0.436				0.28	0.17	0.12	0.097			
	Rich job	0.92	0.13	0.49	0.000**				–0.54	0.14	–0.27	0.000**			
Model 2	Resourceful job	0.32	0.09	0.23	0.000**	0.29	19.81 *df* (4, 195)	0.000**	0.02	0.11	0.02	0.835	0.10	5.36 *df* (4, 195)	0.000**
	Poor job	–0.02	0.14	–0.01	0.91				0.27	0.17	0.12	0.117			
	Rich job	0.85	0.12	0.45	0.001**				–0.54	0.15	–0.27	0.000**			
	SOG	0.28	0.09	0.21	0.000**				–0.03	0.11	–0.02	0.804			
Model 1	Resourceful job	0.37	0.09	0.27	0.00**	0.25	21.98 *df* (3, 196)	0.000**	0.02	0.11	0.01	0.870	0.10	7.16 *df* (3, 196)	0.000**
	Poor job	–0.11	0.14	–0.05	0.436				0.28	0.17	0.12	0.097			
	Rich job	0.92	0.13	0.49	0.000**				–0.54	0.14	–0.27	0.000**			
Model 2	Resourceful job	0.37	0.10	0.27	0.000**	0.25	16.42 *df* (4, 195)	0.000**	0.03	0.11	0.02	0.752	0.12	6.31 *df* (4, 195)	0.000**
	Poor job	–0.11	0.15	–0.05	0.462				0.24	0.17	0.10	0.155			
	Rich job	0.91	0.13	0.48	0.000**				–0.44	0.15	–0.22	0.005**			
	EGI	0.03	0.09	0.02	0.779				–0.19	0.10	–0.14	0.063			
Model 1	Resourceful job	0.37	0.09	0.27	0.000**	0.25	21.98 *df* (3, 196)	0.000**	0.02	0.11	0.01	0.870	0.10	7.16 *df* (3, 196)	0.000**
	Poor job	–0.11	0.14	–0.05	0.436				0.28	0.17	0.12	0.097			
	Rich job	0.92	0.13	0.49	0.000**				–0.54	0.14	–0.27	0.000**			
Model 2	Resourceful job	0.36	0.09	0.26	0.000**	0.26	16.74 *df* (4, 195)	0.000**	0.03	0.11	0.02	0.787	0.10	5.50 *df* (4, 195)	0.000**
	Poor job	0.09	0.15	–0.04	0.549				0.25	0.17	0.11	0.131			
	Rich job	0.89	0.13	0.47	0.000**				–0.51	0.15	–0.26	0.001**			
	CSV	0.09	0.09	0.07	0.315				–0.08	0.10	–0.05	0.460			
Model 1	Resourceful job	0.37	0.09	0.27	0.000**	0.25	21.98 *df* (3, 196)	0.000**	0.02	0.11	0.01	0.870	0.10	7.16 *df* (3, 196)	0.000**
	Poor job	–0.11	0.14	–0.05	0.436				0.28	0.17	0.12	0.097			
	Rich job	0.92	0.13	0.49	0.000**				–0.54	0.14	–0.27	0.000**			
Model 2	Resourceful job	0.31	0.09	0.23	0.000**	0.28	18.60 *df* (4, 195)	0.000**	0.10	0.11	0.07	0.375	0.13	7.42 *df* (4, 195)	0.000**
	Poor job	–0.09	0.15	–0.00	0.952				0.15	-0.17	0.06	0.378			
	Rich job	0.78	0.13	0.41	0.000**				–0.37	0.15	–0.19	0.017*			
	Capset	0.06	0.02	0.19	0.010**				–0.07	0.02	–0.22	0.007**			

UKS, use of knowledge and skills; DKS, development of knowledge and skills; IID, involvement in important decisions; MRW, building and maintaining meaningful relationships at work; SOG, setting own goals; EGI, earning a good income; CSV, contributing to something valuable; Capset, capability set.

*p < 0.05; **p < 0.01.

Concerning work engagement as dependent variable, Table 6 shows that the second model of each multiple regression analysis (including JD-R profiles and capabilities as predictors) was statistically significant in the following cases: JD-R profiles and using knowledge and skills (β_Resourceful_ _job_ = 0.27, *p* = 0.000; β_Rich_ _job_ = 0.47, *p* = 0.000; β_Use_ _of_ _knowledge/skills_ = 0.14, *p* = 0.031); JDR profiles and developing new knowledge and skills (β_Resourceful_ _job_ = 0.26, *p* = 0.000; β_Rich_ _job_ = 0.47, *p* = 0.000); JD-R profiles and involvement in important decisions (β_Resourceful_ _job_ = 0.26, *p* = 0.000; β_Rich_ _job_ = 0.47, *p* = 0.000); JD-R profiles and building and maintaining meaningful work relationships (β_Resourceful_ _job_ = 0.24, *p* = 0.000; β_Rich_ _job_ = 0.45, *p* = 0.000; β_Meaningful_ _work_ _relationships_ = 0.15, *p* = 0.030); JD-R profiles and setting own goals (β_Resourceful_ _job_ = 0.23, *p* = 0.000; β_Rich_ _job_ = 0.45, *p* = 0.000); (β_Setting_ _own_ _goals_ = 0.21, *p* = 0.000); JD-R profiles and earning a good income (β_Resourceful_ _job_ = 0.27, *p* = 0.000; β_Rich_ _job_ = 0.48, *p* = 0.000); and JD-R profiles and contributing to something valuable (β_Resourceful_ _job_ = 0.26, *p* = 0.000; β_Rich_ _job_ = 0.47, *p* = 0.000). The JD-R profiles and the capability set statistically significantly predicted work engagement (β_Resourceful_ _job_ = 0.23, *p* = 0.000; β_Rich_ _job_ = 0.41, *p* = 0.000; β_Capability_ _set_ = 0.19, *p* = 0.000). Based on these findings, hypothesis 2 is accepted.

Concerning intention to leave as dependent variable, Table 6 shows that the second model of each multiple regression analysis (including JD-R profiles and capabilities as predictors) was statistically significant in the following cases: JD-R profiles and using knowledge and skills (β_Rich_ _job_ = −0.25, *p* = 0.001; β_Use_ _of_ _knowledge/skills_ = −0.22, *p* = 0.002); JD-R profiles and developing new knowledge and skills (β_Rich_ _job_ = −0.24, *p* = 0.000); JD-R profiles and involvement in important decisions (β_Rich_ _job_ = −0.22, *p* = 0.000); JD-R profiles and building and maintaining meaningful work relationships (β_Rich_ _job_ = −0.25, *p* = 0.000); JD-R profiles and setting own goals (β_Rich_ _job_ = −0.27, *p* = 0.000); JD-R profiles and earning a good income (β_Rich_ _job_ = −0.22, *p* = 0.005); and JD-R profiles and contributing to something valuable (β_Rich_ _job_ = −0.26, *p* = 0.001). The JD-R profiles and the capability set statistically significantly predicted turnover intention (β_Rich_ _job_ = −0.19, *p* = 0.017; β_Capability_ _set_ = −0.22, *p* = 0.007). Based on these findings, hypothesis 3 is accepted.

## Discussion

This study utilized the JD-R model (Demerouti et al., 2001; Lessener et al., 2019) to examine the association between JD-R profiles (based on workload, emotional load, intrinsic job characteristics, supervisor relations, remuneration, and performance support), capabilities of SETs, and work engagement and intention to leave as functionings. Latent profile analysis identified four JD-R profiles: resourceful jobs, demanding jobs, poor jobs, and rich jobs. JD-R profiles were associated with the capabilities of SETs. Together, JD-R profiles and the capability set predicted a large percentage of the variance in work engagement and a moderate percentage of the variance in intention to leave.

In line with Van den Broeck et al.’s (2011) findings, four JD-R profiles were identified through latent profile analysis: the resourceful, demanding, poor, and rich job. The resourceful job featured low demands and moderate to high resources, while moderate demands and low resources characterized the demanding job. The poor job had high demands and low resources. Lastly, the rich job was characterized by moderate demands and high resources.

Job demands-resources profiles were associated with capabilities. The results showed that a poor job resulted in a low capability to use knowledge and skills. In contrast, resourceful and rich jobs predicted capabilities to develop new knowledge and skills, be involved in important decisions, to build and maintain meaningful relationships, and set their own goals. A poor job negatively affected capabilities to build and maintain meaningful relationships, set own goals, and contribute to something valuable. A rich job predicted the capability to earn a good income and to contribute to something valuable. Interestingly, the results indicated that SETs with less job tenure (compared to those with more tenure) were more inclined to show capabilities regarding the use of knowledge and skills, the development of new knowledge and skills, involvement in important decisions, earning a good income, and contributing to something valuable.

As was expected based on the SE model (e.g., Van der Klink et al., 2016; Van der Klink, 2019), the capabilities SETs were associated with their work engagement and intentions to leave. The following capabilities were associated with work engagement: the use of knowledge and skills, development of knowledge and skills, involvement in important decisions, building and maintaining meaningful relationships at work, setting own goals, earning a good income, and contributing to something valuable. Furthermore, the use of knowledge and skills, development of knowledge and skills, involvement in important decisions, building and maintaining meaningful relationships at work, contributing to something valuable, and earning a good income were associated with SETs’ intentions to leave.

Job demands-resources profiles and specific capabilities were associated with the work engagement of SETs. More specifically, the results showed that resourceful and rich jobs combined with each of the following capabilities predicted large percentages of the variances (varying from 25% to 28%) in work engagement: using knowledge and skills, developing new knowledge and skills, being involved in important decisions, building and maintaining meaningful work relationships, setting own goals, earning a good income, and contributing to something valuable. Resourceful and rich jobs and the capability set also predicted a large percentage of the variance in work engagement. These findings confirmed that resourceful and rich jobs, characterized by moderate workload, and resources such as job characteristics, supportive supervisory relationships, salary, and performance support mattered for work engagement. However, the capabilities of SETs also mattered for their work engagement. Moreover, SETs in these JD-R profiles (compared to demanding and poor jobs) experienced lower emotional demands. Although Minghui et al. (2018), Murangi and Bailey (2022) conducted variable-oriented (rather than person-oriented) studies, their findings showed that job demands and resources (e.g., autonomy, colleague support and rewards, and recognition) were associated with work engagement.

Concerning intention to leave as a dependent variable, the results showed that the absence of a rich job and each of the following capabilities predicted moderate percentages of the variance: use of knowledge and skills, development of new knowledge and skills, involvement in important decisions, building and maintaining meaningful work relationships, setting own goals, earning a good income, and contributing to something valuable. Moreover, a rich job and the capability set of SETs predicted low intentions to leave. These results showed that a rich job, accompanied by a capability set (and specific capabilities), mattered for the retention of SETs.

Interestingly, JD-R profiles (specifically resourceful and rich jobs) were better predictors of work engagement than intentions to leave. Resourceful and rich jobs were characterized by low to moderate job demands and the availability of high job resources. Research confirms that the more job resources there are in a work context (compared to job demands), the more engaged employees will be (Albrecht et al., 2021). This finding might be explained by the lack of mobility of SETs in Namibia. Having fewer options for employment tends to make people less likely to leave their jobs (Shapira-Lishchinsky, 2012). Therefore, SETs might have lower intentions to leave because of the lack of opportunities in the Namibian education sector and labor market.

Job demands (e.g., pace, workload, mental, and emotional load) can facilitate or inhibit functioning. If demands are aligned with what SETs value, they will be more engaged at work and less likely to leave (Van der Klink, 2019). Conversely, they may suffer from disengagement and leave if these demands are not valued. The findings indicated that an ideal job environment for a special education teacher in Namibia would be a rich job characterized by moderate job demands and high job resources. Regarding overload, SETs in rich jobs did not differ much from those in poor jobs, although SETs in poor jobs experienced more emotional demands than those in rich jobs. However, rich and poor jobs differed most in terms of the availability of job resources, such as experiences of the nature of the job, supervisory support, salary, and performance support (in line with findings of variable-oriented studies, e.g., Minghui et al., 2018; Brunsting et al., 2022; Murangi and Bailey, 2022). Indeed, job autonomy and supervisor support as resources might play a role in converting emotional demands to values (e.g., developing new knowledge and skills or contributing something valuable to society).

Furthermore, the capabilities of teachers, including involvement in important decisions, developing knowledge and skills, building and maintaining meaningful relationships at work, setting their own goals, earning a good income, and contributing to something valuable, are critical. Unfortunately, however, few teachers were in the rich job profile. These findings support the usefulness of the SE framework. It seems essential to consider capabilities based on values, opportunities, and achievements (Abma et al., 2016; Van der Klink et al., 2016; Van der Klink, 2019). A rich JD-R profile is essential to develop and optimize teacher capability and enhance teachers’ work engagement. Zimba et al. (2013) found that teachers in Namibia had to deal with heavy administration work, large classrooms, a lack of teaching materials, and limited learning and training opportunities. According to Dehaloo and Schulze (2013), teachers face heavy workloads, poor remuneration, and limited learning and career advancement opportunities, which negatively affect their work engagement. Specifically, in developing countries where poverty and vast inequalities exist, it has proven challenging to provide adequate resources to help teachers function effectively (Ncube and Hlatywayo, 2014).

Teachers in demanding and poor jobs will become less engaged and more inclined to leave, placing their sustainable employability at risk. The current study results indicated that more than half (*n* = 104) of SETs in Namibia experienced demanding or poor jobs. This is disastrous for learners with disabilities who need special education schools to become capable citizens, their parents, and the Ministry of Education, Arts, and Culture in Namibia.

Billingsley and Bettini (2019) raised a question about the type of demands that will affect SETs adversely. The study’s results showed that emotional demands played a larger role than pace and amount of work in predicting disengagement and intentions to leave, whereas job demands played a smaller role. High emotional demands, overload, and a lack of resources interfere with teachers’ functioning (e.g., work engagement and intention to leave). Moreover, job demands and resources were associated with SETs’ work engagement and intentions to leave. Teachers in rich jobs (with moderate demands, high resources) and resourceful jobs (with low job demands, moderate job resources) were more engaged compared to those in demanding jobs (with moderate job demands, low job resources). However, it seems that it was not only the combination of job demands and resources that mattered; the capabilities of teachers also played a vital role. Specific capabilities that mattered for teachers’ work engagement included using existing knowledge and skills, meaningful work relations, and setting own goals. However, more than specific capabilities, it was evident that the capability set played a significant positive role in the work engagement of SETs.

Concerning retention, the results showed that a combination of job demands and resources explained a moderate percentage of the variance in the SETs’ intentions to leave (as suggested by Billingsley and Bettini, 2019). Teachers in the rich JD-R profile (compared to the demanding profile) had significantly lower intentions to leave. However, in addition to the JD-R, a lack of two specific capabilities, namely using existing knowledge and skills and earning a good income, also contributed to intentions to leave. Moreover, the capability set JD-R profiles explained a moderate percentage of the variance in SETs’ intentions to leave.

To sustain quality special education provision in Namibia, the focus must be on enhancing and building the capabilities of SETs. Interventions should focus on all capabilities to develop and strengthen the capability set (which consists of values, enablement, and achievement). To facilitate the work value development of teachers (and individuals who are trained as teachers), managers should question their core assumptions about human nature and understand how their mental models affect their managerial practices (Heil et al., 2000; Grant, 2021). Efficient and effective human resource management practices (e.g., recruitment and selection, induction, training and development, coaching and mentorship, occupational health and wellbeing, performance management and remuneration) are vital to building the three dimensions of capabilities (i.e., values, enablement, and achievement). In the performance management process, managers and teachers must understand the seven capabilities and communicate about values, enablement, and achievement.

The resources of special education schools must be upgraded (Billingsley and Bettini, 2019). A balance of job demands and resources does not imply that individuals are engaged and will not quit their jobs. As suggested by the capability approach, resources are inadequate indicators of wellbeing since individuals have different needs for resources and different abilities to convert them into functionings. Work engagement and retention of SETs require more than just resources; capabilities of teachers should be developed to counteract challenges such as the large classrooms, the lack of assistive teaching and learning devices, high workloads, the shortage of teachers, and a lack of teaching materials (Haihambo, 2004; Murangi and Bailey, 2022). It is essential to assess how job demands and resources affect teachers’ capabilities and implement interventions directed at workload, role clarity, colleague support, supervisor relations, and providing resources (Buckingham and Goodall, 2019; Rothmann and Cooper, 2022). Many SETs are in poor and demanding jobs. Van den Broeck et al. (2011) assert that increasing job resources in the poor and demanding job profiles may benefit employees. Teachers should be enabled by crafting resourceful and rich jobs. Chitiyo et al. (2019) found that SETs in Namibia needed professional development regarding behavior management, learning strategies, diversity management, instructional methods, assessment, knowledge of types of disabilities, teaching life skills, and collaboration with parents. Investing in job resources and capabilities of SETs can contribute to their work engagement and retention.

## Limitations and recommendations for future research

Several limitations were encountered in this study. Firstly, only 200 SETs, from a total of 300, participated in the study due to the voluntary nature of the study, coupled with COVID-19 restrictions. In addition, teachers had a lot of teaching content to cover due to the unforeseen lockdowns. As such, teacher time was limited. Therefore, it is impossible to generalize the findings to all SETs in Namibia. Future studies must be cognizant of periods in the year when constraints on teacher time are fewer to reach more teachers. Secondly, while using a cross-sectional survey in this study was helpful in cost-effectively exploring the relations between constructs (Spector, 2019), it is impossible to prove the causality of predictions. A longitudinal study will help uncover patterns in SETs job demands and resources, capabilities, and functioning over time. Thirdly, an existing measure of work values was used (Abma et al., 2016). However, unique work values might exist in Namibia. In the future, cross-cultural qualitative studies might be valuable to investigate whether more African-specific work values should be included in a capability measure. Billingsley and Bettini (2019) also recommended that high-quality qualitative methods be used to study specific functionings (such as the retention of SETs). Fourthly, although interactions between job demands and resources were considered (by using latent profile analysis) in this study, future studies might focus on the interactions between job demands and resources and capabilities in a variable-oriented study to capture the complex interrelationships between variables that impact work engagement and intentions to leave of SETs (see Billingsley and Bettini, 2019). Fifthly, more female SETs (68.5%) participated in the study. The present study did not investigate the reasons for the dominance of females in the SET profession. Future studies should investigate the role of gender concerning capabilities and functioning teachers in the SET profession.

## Conclusion

This study investigated the effects of JD-R profiles and capabilities on two functionings (engagement and intention to leave). The study provided insight into the JD-R profiles that predicted the capabilities of SETs in Namibia. The JD-R profiles created for this study indicated that the ideal job profile, which could significantly impact engagement and reduce turnover intentions, was the rich job profile. The question that needs to be investigated by all stakeholders is what strategies can be employed to create rich jobs in Namibian special schools to promote the sustainable employability of SETs.

The person-oriented approach employed to study the combined effects of job demands and resources on the capabilities of SETs resulted in valuable insights into their functionings. The distinct combinations of demands and resources should inform policies and practices that facilitate capability identification, development, and optimal functioning of SETs in Namibia.

## Data availability statement

The dataset presented in this study can be found in the following repository: Rothmann and Muningua (2022).

## Ethics statement

The studies involving human participants were reviewed and approved by the Economic and Management Sciences Research Ethics Committee, North-West University, South Africa. The participants provided their written informed consent to participate in this study.

## Author contributions

AM took the lead in conceptualizing and writing the manuscript and collected and analyzed the data. SR conducted the data analyses, acted as an additional writer, and reviewed the manuscript. MN acted as an additional writer and reviewed the manuscript. All authors contributed to the article and approved the submitted version.

## Footnotes

1 Given three educational options for learners with disabilities in Namibia, the term special education teacher is used in this study to refer to teachers who deliver quality education to learners with disabilities in special (resource), special classes in mainstream schools, and inclusive schools (see Republic of Namibia, Ministry of Education, 2018).

2
http://www.moe.gov.na/
